# Hacking into bacterial biofilms: a new therapeutic challenge

**DOI:** 10.1186/2110-5820-1-19

**Published:** 2011-06-13

**Authors:** Christophe Bordi, Sophie de Bentzmann

**Affiliations:** 1 Laboratoire d'Ingénierie des Systèmes Macromoléculaires, UPR9027 CNRS - Aix Marseille Université, Institut de Microbiologie de la Méditerranée, 31 Chemin Joseph Aiguier, 13402 Marseille, France

## Abstract

Microbiologists have extensively worked during the past decade on a particular phase of the bacterial cell cycle known as biofilm, in which single-celled individuals gather together to form a sedentary but dynamic community within a complex structure, displaying spatial and functional heterogeneity. In response to the perception of environmental signals by sensing systems, appropriate responses are triggered, leading to biofilm formation. This process involves various molecular systems that enable bacteria to identify appropriate surfaces on which to anchor themselves, to stick to those surfaces and to each other, to construct multicellular communities several hundreds of micrometers thick, and to detach from the community. The biofilm microbial community is a unique, highly competitive, and crowded environment facilitating microevolutionary processes and horizontal gene transfer between distantly related microorganisms. It is governed by social rules, based on the production and use of "public" goods, with actors and recipients. Biofilms constitute a unique shield against external aggressions, including drug treatment and immune reactions. Biofilm-associated infections in humans have therefore generated major problems for the diagnosis and treatment of diseases. Improvements in our understanding of biofilms have led to innovative research designed to interfere with this process.

## Review

### Inside biofilms

Biofilm notion is based on single-celled unicellular individuals (bacteria, fungi, or yeasts) forming a sedentary community within a complex structure, displaying spatial and functional heterogeneity [1]. Bacterial biofilms account for a particular problem for human health, because they are responsible for several infectious diseases, associated with many inert surfaces, including medical devices for internal or external use. They are additionally suspected to be present in hospital water networks and as reservoirs may lead to subsequent acquired infections after patients' hospitalization.

The presence of biofilms is probably underestimated, principally because of the need for *in vivo *diagnosis [2]. Early studies described biofilms as an aspect of microbial physiology [3], which almost all bacterial species can adopt. The multicellular structure of the biofilm makes it possible for the bacteria concerned to undergo dormancy and hibernation, enabling them to survive and to disseminate their genomes. It may therefore be considered as a step in the bacterial cell cycle.

Biofilms also display unique properties, such as multidrug tolerance and resistance to both opsonization and phagocytosis, enabling them to survive in hostile environmental conditions and to resist selective pressures [4]. It seems that host immunity is totally ineffective at clearing these microcommunities and evidence has been obtained that shows that immune cells are paralyzed with impeded phagocytosis capacities [5] or decreased burst response after phagocytosis with lowered production of reactive oxygen species [6]. This community also is unique in that it brings together different species in a structure in which they can cooperate, rather than compete. The biofilm thus constitutes a microbial society, with its own set of social rules and patterns of behavior, including altruism and cooperation, both of which favor the success of the group [7,8] with task-sharing behavior, on the one hand, and competition [9], on the other. Certain subpopulations may display specialization. All of these patterns of behavior are orchestrated by communication, which may be chemical or genetic [10]. The biofilm thus constitutes a unique way to stabilize interactions between species, inducing marked changes in the symbiotic relationships between them and affecting the function of the microbial community [11].

**Figure 1:**
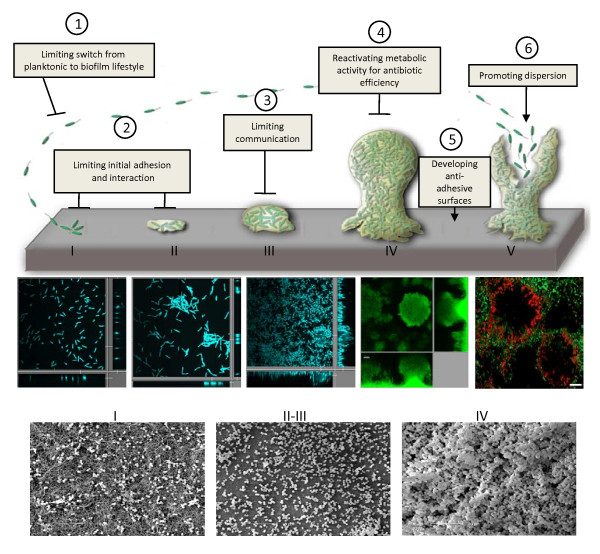
**Temporal evolution of biofilm**. Schematization of the four-stage universal growth cycle of a biofilm with common characteristics, including initiation (I), maturation (II and III), maintenance (IV), and dissolution (V). Steps in *P. aeruginosa *are presented labelled with DAPI (A-C), chromosomal GFP (D) (personal data), or LIVE/DEAD BacLight kit (E) (Boles et al., 2005), observed with confocal microscopy and in *S. aureus *(F-H) in scanning electron microscopy (personal data). Potential hacking strategies are presented, including limiting 1) switch from planktonic to biofilm lifestyle (protein engineering of key players including c-di-GMP proteins, global regulators), 2) initial adhesion and interaction (glycomimetics), 3) communication (compounds interfering with QS autoinducers), 4) reactivating metabolic activity for increasing antibiotic efficiency (iron chelating procedure as an adjunct to conventional antibiotics), 5) developing anti-adhesive surfaces (silver or antiseptic-coated surfaces for endotracheal tubes), and 6) promoting dispersion (NO, capsules or dispersin-like molecules, phages).

This multicellular arrangement also creates chemical and metabolite gradients and heterogeneity in oxygen availability. As such, it is a potentially stressful environment for aerobic species, necessitating adaptation to oxygen paucity [12]. Starvation is an important trigger of stress responses [13] and is associated with changes in metabolic and catabolic pathways and with signs of membrane stress [14,15]. Stressful associated conditions in biofilms represent a unique way to generate genetic diversity additionally and to drive evolution. The emergence of new subpopulations, such as small-colony variants (genetic or adaptative diversification), persisters, and cells tolerant to imposed constraints, represents a new challenge for microbiologists, who will need to develop an integral, holistic view of the community. Common biofilm properties have been defined, such as the need for a substrate and "preconditioning surfaces," the specialization of subpopulations (known as "division of labor" [16]), the production of a hydrated matrix shaping the community, and the division of this life cycle into stages (Figure 1).

Biofilms, like other communities, form gradually over time. Whatever the bacterial species involved and the complexity of the resulting community, biofilm formation is a dynamic process highly dependent on environmental signals, passing through a four-stage universal growth cycle consisting of initiation, maturation, maintenance, and dissolution phases, regardless of the phenotype of the constituent microorganisms [17]. Despite some common traits, generalizations cannot be made in particular when considering that it mostly associates multiple species.

### Why are biofilms difficult to treat?

Biofilms in vivo are very difficult to diagnose essentially due to the lack of sampling methods and markers, but bacterial cell clusters in discrete areas in the host tissue associated with host inflammatory cells can signal such biofilm infections [18]. Chemical, physiological, and genetic heterogeneity of the embedded bacterial population increases over both space and time [19] (Figure 1). This has been observed in *Staphylococcus aureus *biofilms, in which cells exist in at least four distinct states: aerobic growth, fermentative growth, dead, and dormant [20].

Multidrug resistance, more than any other property of biofilms, provides a clear demonstration that population behavior is not the sum of the contributions of single cells. Biofilms are unique multicellular constructions of bacteria from one or several species, in which horizontal genetic transfer may occur easily, thus facilitating crossbreeding of resistance genes. The bacteria within biofilms are embedded in a matrix of exopolysaccharides (EPS) that they produce themselves. This matrix limits antibiotic diffusion. The association of molecules of various types within the biofilm, including EPS and DNA, constitutes a physical barrier to the diffusion of antimicrobial agents. However, many studies have surprisingly shown that the penetration of antibiotics is not limited in bacterial biofilms. For example, fluoroquinolones diffuse rapidly within *Pseudomonas aeruginosa *[21] and *Klebsiella pneumoniae *[22] biofilms, tetracycline diffuses rapidly in *Escherichia coli *biofilms [23], and vancomycin diffuses rapidly in *Staphylococcus epidermidis *biofilms [24]. Aminoglycosides are the only molecules for which poor penetration has been reported in biofilms of an alginate (the mucoid EPS)-producing strain of *P. aeruginosa *[25]. As EPS differ considerably between, and even within species, the limited diffusion of antimicrobial drugs within bacterial biofilms certainly has been underestimated. Regulation of specific drug resistance-associated genes due to unique environmental stresses or starvation conditions can be observed in bacterial biofilms. These conditions may favor the emergence of dormant cells called persisters [26].

Persisters are in a particular physiological state with low levels of translation but a unique gene expression profile [27], associating the switch off of genes encoding metabolic proteins together with operons encoding toxin-antitoxin pairs switched on. The latter probably play a role in competition in addition to contribute to dormancy. However, persister cells, which are resistant to killing by antibiotics and can survive drug treatment, account for only a small proportion of the biofilm population [28]. Indeed, when dispersed mechanically, most biofilm cells seem to be as susceptible to inhibitors as planktonic cells. A number of cells are drug-tolerant because of their particular physiological state in the biofilm, due to nutrient and oxygen limitation, for example. Some resistance mechanisms may be stronger in biofilms, given that specific efflux pumps have been shown to be more efficient in *P. aeruginosa *[29] and *E. coli *[30] biofilms. However, this mechanism is not universal, and some efflux pump inhibitors can reduce or even abolish *E. coli *biofilm formation [31].

A novel mechanism of biofilm-associated antibiotic resistance has been described recently in *P. aeruginosa*: released DNA, the highly anionic polymer working as a cation chelator in the extracellular matrix, creates a localized cation-limited environment. This cation-starvation is detected by *P. aeruginosa*, leading to the induction of LPS modification genes and resistance to antimicrobial drugs, such as cationic antimicrobial peptides and aminoglycosides [32]. Another interesting biofilm-specific resistance mechanism also has been identified in this bacterium. The biofilm *ndvB*-dependent production of glucans in the periplasm leads to aminoglycoside sequestration in this cellular compartment, preventing them from reaching their ribosomal targets; this mechanism is unique to *P. aeruginosa biofilms *[33]. In multimicrobial biofilms containing *Candida albicans *and *S. aureus*, such as that occurring on the surface of indwelling medical devices, resistance of *S. aureus *to vancomycin is higher in the polymicrobial biofilm. This increased resistance of *S. aureus *requires viable *C. albicans *and is mediated in part by the *C. albicans *matrix [34], although *C. albicans *growth and sensitivity to amphotericin B are not altered in the polymicrobial biofilm.

It is now widely accepted that life in a sedentary community confers a unique type of bacterial resistance, known as biofilm-associated antimicrobial resistance. This resistance is highly problematic for effective therapeutic decisions, especially when considering that many resistance phenotypes are shut down when bacterial samples are isolated from patients and examined for clinical bacteriological phenotypes.

### How are biofilms built?

Our understanding of the molecular basis of bacterial biofilm development has benefited from improvements in genetics, genomics, and the development of visualization techniques unraveling the processes involved in biofilm development, physiology, and adaptation. A plethora of systems that enable bacteria to identify and anchor themselves onto appropriate surfaces, to stick to each other, to construct multicellular communities several hundreds of micrometers thick, and to detach from the community has been identified and characterized in many biofilm-forming bacteria (Figure 1). It is not possible to identify general molecular profiles for a given bacterial species, because some genes are important for biofilm formation under both static and dynamic conditions, whereas others are important only under dynamic biofilm conditions [35]. However, throughout the bacterial kingdom, these genes can be separated into those encoding appendages consisting of oligomerized subunits responsible for motility (type IV pili or TFP, flagella) or with other functions (fimbriae, other types of pili, curli), EPS, surface adhesins, or other secreted elements. The molecular machines responsible for assembling or secreting these systems are, of course, highly dependent on the simple or double membranes of Gram-positive and Gram-negative bacteria, respectively [36,37]. Their role in biofilm initiation and structuring also is highly dependent on environmental conditions and the surfaces encountered by the bacteria [38]. Each bacterial species has its own adhesion toolkit, containing a number of molecules, different for each species that may be used antagonistically or synergistically, depending on the situation with which the bacterium is faced. Global expression at the patient bed is required to understand how bacteria form biofilms in patients, especially when considering that *in vivo *bacterial situations can widely differ with *in vitro *behavior [39].

### What signals trigger biofilm structuration?

**Figure 2:**
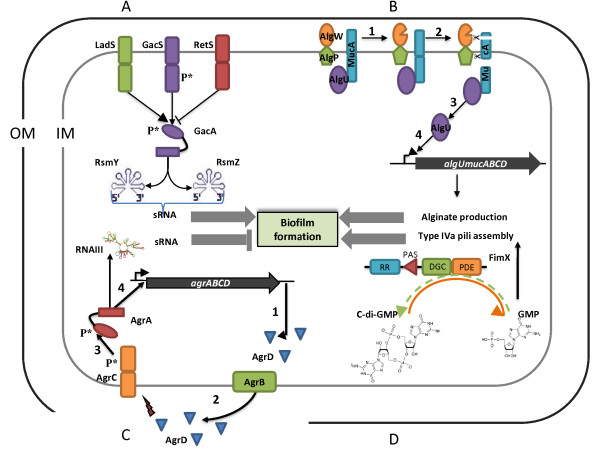
**Regulatory networks controlling transition between planktonic and biofilm lifestyle**. The external frames illustrate the bacterial envelope with one or two membranes (OM: outer membrane, IM: inner membrane) according to Gram-positive (C) and Gram-negative bacteria (A, B, and D), respectively. **A **Control of biofilm formation in *P. aeruginosa *through the TCS GacS (HK)/GacA (RR) mediated by sRNA *rsmY and rsmZ *gene transcription and modulated by RetS and LadS, two additional HK in *P. aeruginosa*. **B **Control of EPS alginate in *P. aeruginosa*, which further impacts biofilm architecture by the system ECF sigma factor AlgU - anti-sigma MucA - AlgP (IM)-AlgW (periplasmic) complex: 1) activation of AlgW/AlgP, 2) cleavage of MucA, 3) release of AlgU, 4) activation of the *alg UmucABCD *operon. **C **Control of *S. aureus *biofilm formation through the Agr QS system: 1) AgrD autoinducer production, 2) AgrD autoinducer accumulation in the extracellular medium where it reaches a threshold, 3) activation of the TCS AgrCA by AgrD at the threshold concentration, 4) AgrA-dependent activation of the sRNA RNA III expression repressing expression of genes involved in biofilm formation together with amplification loop of *agrABCD*. **D **Control of *P. aeruginosa *biofilm formation through the intracellular second messenger c-di-GMP level controlled by the FimX protein having DGC and PDE domains, a RR domain, and a PAS domain. Note that in FimX protein only PDE activity is detectable (continuous arrow), whereas DGC activity is undetectable (dotted arrow).

Biofilm formation is highly dependent on regulatory networks governing the switch between planktonic and sedentary lifestyles. These networks integrate environmental signals through adequate sensing systems triggering appropriate responses, including two-component and ECF signaling pathways, quorum sensing (a multicellular response) resulting in the production of autoinducers, which are small diffusible molecules [40,41] and other molecules, including c-di-GMP [42] (Figure 2). The stepwise formation of the biofilm, such as developmental processes, involves the switching on of a specific genetic program, leading to coordinated patterns of gene expression and protein production.

Two-component system (TCS) and extracytoplasmic function (ECF) signaling pathways are the major signaling mechanisms used by bacteria to monitor external and internal stimuli (e.g., nutrients, ions, temperature, redox states) and translate these signals into adaptive responses. The TCS pathways (Figure 2A) include two proteins: a histidine kinase (HK) protein, called "sensor," and a cognate partner, called "response regulator" (RR). Upon detection of the stimulus, the HK is activated and auto-phosphorylates on a conserved histidine residue. The phosphoryl group is then transferred onto a conserved aspartate residue on the cognate RR [43]. Phosphorylation results in RR activation, which is most frequently a transcriptional regulator. As an example, the GacS (HK)/GacA (RR) TCS is one of the major systems involved in the control of *P. aeruginosa *biofilm formation. Once activated by an unknown signal, the GacS/GacA TCS switches on the transcription of the *rsm *genes. The *rsm *genes encode two small non-coding RNA (sRNA), RsmY and RsmZ, of which the expression level is a key player in controlling switch between planktonic and biofilm lifestyles [44]. High expression of *rsmY *and *rsmZ *leads to high biofilm formation, whereas a reduced expression of them is associated with an impaired biofilm formation. The Gac regulatory pathway has been linked to two additional HK RetS and LadS. Although RetS has been demonstrated to antagonize GacS, thus repressing genes required for biofilm formation [45], LadS reinforces GacS-dependent activation of genes required for biofilm formation [46]. In parallel, the Gac system activates antibiotic resistance toward aminoglycosides (gentamicin and amikacin) and chloramphenicol [47], thus linking once more biofilm lifestyle and antimicrobial resistance. TCS-dependent regulation of biofilm formation is widespread in many bacteria as illustrated by the positive control exerted by the GraS (HK)/GraR (RR) TCS on *S. aureus *biofilm induction [48].

The second major signaling mechanism used by bacteria and probably underestimated is the ECF signaling pathway (Figure 2B), which involves an alternative sigma factor, an anti-sigma factor located preferentially in the cytoplasmic membrane, sequestering and inhibiting its cognate sigma factor [49] and one or several periplasmic or outer membrane proteins required for the activation of the pathway [50]. Upon perception of the extracellular signal by the periplasmic or outer membrane proteins, degradation of the anti-sigma factor induces releasing of the sigma factor, which can promote the transcription of a specific set of target genes. In *P. aeruginosa*, for example, AlgU ECF sigma factor controls production of the EPS alginate, which further impacts biofilm architecture [51]. The AlgU sigma factor functions with the anti-sigma MucA, which C-terminal periplasmic domain is cleaved by the AlgW protease in response to an unknown signal [52].

Bacteria also use a multicellular response to coordinate expression of genes required for biofilm in a population density-dependent manner, called quorum sensing (QS) (Figure 2C), defined as a bacterial communication process utilizing small, diffusible molecules termed autoinducers or pheromones. Autoinducers are different between Gram-negative and Gram-positive bacteria, using preferentially N-acyl-homoserine lactones and oligopeptides, respectively. Autoinducers accumulate outside reflecting the growing population density and, upon reaching a concentration threshold, regulate virulence and pathogenicity genes. Detection of autoinducer threshold may utilize a HK, or autoinducers can enter passively or actively the cell and bind a regulator protein, both combinations trigger a specific genetic response [53]. In S. *aureus*, transition between planktonic and biofilm lifestyles is predominantly controlled by QS. The *S. aureus *QS is encoded by the *agr *operon where *agrD *encodes the autoinducer. Once produced, exported, and present at the threshold concentration, AgrD pheromone controls through the TCS AgrCA, the expression of the small non-coding RNA, RNAIII; thus RNAIII down-regulates genes encoding adhesins required for biofilm formation [54,55]. It thereby promotes dispersion together with extracellular protease activity [56], linking inversely the bacterial population size and biofilm formation together with probable resistance to glycopeptides antibiotics [57]. Most glycopeptide-resistant *S. aureus *are *agr *dysfunctioning even though the link between *agr *function and glycopeptide resistance is still debated.

Finally, among signaling molecules is the intracellular second messenger c-di-GMP (Figure 2D). The amount of this messenger is tightly controlled, being increased by the activity of diguanylate cyclases (DGC) carrying GGDEF domains and decreased by the activity of phosphoesterases (PDE) carrying EAL domains. In bacteria, high c-di-GMP levels are generally associated with the stimulation of biofilm formation via the production of adhesive surface organelles or EPS synthesis and a decrease in motility.

Many proteins containing GGDEF or EAL domains are linked to various N-terminal sensory input domains, suggesting that several signals from the environment are integrated through the c-di-GMP signaling pathway. In *P. aeruginosa*, the FimX protein controls expression of genes encoding Type IVa pili involved in early step of biofilm formation [58]. FimX possesses both imperfect DGC and PDE domains; however, only the PDE activity is detectable. The FimX protein is associated with a RR domain and a PAS domain; the latter is probably involved in sensing oxygen and redox potential [59]. These signals are potentially the activating signals.

The regulatory networks controlling transition between planktonic and biofilm lifestyle are far from being elucidated and involve intricate crosstalk between regulatory pathways. These networks require extensive genetic studies to understand how bacteria integrate signals from the environment to establish into multicellular communities.

### Where can we hack?

Because this biofilm lifestyle may be associated with human infectious diseases and account for 80% of bacterial chronic inflammatory and infectious diseases, several lines of research are currently focusing on the possibility of hacking into biofilm initiation, structuration or communication, and promoting dispersion [60] (Figure 1), even though we are far from understanding the complex genetic basis for biofilm formation *in vivo*.

Undoubtedly, due to antimicrobial tolerance, slow-growing cells, and EPS matrix, biofilm-associated infections do not respond consistently to therapeutically achievable concentrations of most antimicrobial agents. Practicians, therefore, must integrate these notions to direct clinical decision and further adapt antimicrobial therapy to such types of combined infectious conditions. This is particularly successful when antimicrobial lock technique (ALT) is applied in particular to combat bacterial biofilms on central veinous catheters [61,62]. This technique corresponds to an instillation of antimicrobial drugs with bactericidal rather than bacteriostatic properties in the catheter *in situ *for a sufficient dwell time and at high concentrations (mg/ml). However, for most studies that evaluate ALT in patients, true elimination of bacterial biofilms has not been checked and treatment success has been based on negative culture results of blood samples or absence of clinical symptoms in patients. Because very high doses of antimicrobials are recommended, ALT can induce secondary antimicrobial resistance and potential toxicity for the patient [62]. This technique has been tested with several other molecules, such as chelating agents, ethanol, and taurolidine-citrate and gives promising results for reduced incidence of biofilms on central-venous access devices in human studies [62].

Additionally, all new information concerning the functioning of biofilms may potentially lead to strategies for dismantling this microbial community [63] and actually requires to be validated *in vivo*. Much effort has focused on compounds interfering with QS autoinducers [64], molecules enhancing dispersion, such as NO, capsules or dispersin-like molecules and, recently, phages [65]. Altering general regulatory pathways by protein engineering of key players also are very promising tracks (e.g., c-di-GMP proteins, global regulators) [60,66]. For example, interfering with DGC protein activity and therefore with c-di-GMP biosynthesis would represent a promising track [67]. Sulfathiazole is a sulfonamide that has been identified as the sole anti-biofilm molecule against *E. coli *strains and acts indirectly on c-di-GMP levels by targeting nucleotide synthesis rather than on DGC activity. Because anaerobic growth within biofilms could depend substantially on iron availability and is critical for biofilm-associated antimicrobial resistance, iron chelation has been proposed as an adjunct to conventional antibiotics, such as aminoglycoside administration to disrupt variable-aged *P. aeruginosa *biofilms [68,69]. Increasing efforts have been dedicated to molecules interfering with adhesive structures and to the development of new surfaces for internal or external medical devices [70]. This is illustrated by the recent demonstration of broad and high-level antimicrobial activity *in vitro *of antiseptic-coated as well as silver-coated endotracheal tubes to prevent adherence and biofilm formation of drug-resistant bacteria (MRSA, MDR *P. aeruginosa*, MDR *Acinetobacter baumannii*, ESBL *K. pneumoniae*, and MDR *Enterococcus cloacae*) and yeasts (*C. albicans*) causing ventilator-associated pneumonia (VAP) in critically ill patients. The promising development of antiseptic-coated devices requires additional animal studies and prospective, randomized clinical trials to evaluate whether they potentially induce emergence of bacterial resistance and reduce the risk of VAP in critically ill patients [71].

## Conclusions

There is a very dynamic research activity in the biofilm field, especially because this bacterial lifestyle may be associated with human infectious diseases. However, the *in vivo *biofilms are far more complex than those studied *in vitro *due to the underestimation of environmental parameters or the numbers of species controlling biofilm formation. Understanding the genetic basis of biofilm formation *in vitro *together with the definition of biofilm signatures *in vivo *in infected patients is a key requirement for efficiently hacking into biofilm strategy.

## Competing interests

The authors declare that they have no competing interests.

## Authors' contributions

CB and SdeB wrote and approved the final manuscript.
